# Iron-Folic Acid Supplementation During Pregnancy Reduces the Risk of Stunting in Children Less Than 2 Years of Age: A Retrospective Cohort Study from Nepal

**DOI:** 10.3390/nu8020067

**Published:** 2016-01-27

**Authors:** Yasir Bin Nisar, Michael J. Dibley, Victor M. Aguayo

**Affiliations:** 1 United Nations Office for Project Services (UNOPS), Diplomatic Enclave, Islamabad 44000, Pakistan; 2 Sydney School of Public Health, The University of Sydney, Sydney 2006, Australia; michael.dibley@sydney.edu.au; 3 United Nations Children’s Fund (UNICEF), Regional Office for South Asia, Kathmandu 5815, Nepal; vaguayo@unicef.org

**Keywords:** Stunting, child, IFA supplementation, risk

## Abstract

The aim of the study was to investigate the effect of antenatal iron-folic acid (IFA) supplementation on child stunting in Nepalese children age <2 years. A retrospective cohort study design was used, in which a pooled cohort of 5235 most recent live births 2 years prior to interview from three Nepal Demographic and Health Surveys (2001, 2006 and 2011) was analysed. The primary outcome was stunting in children age <2 years. The main exposure variable was antenatal IFA supplementation. Multivariate Poisson regression analysis was performed. In our sample, 31% and 10% of Nepalese children age <2 years were stunted and severely stunted, respectively. The adjusted relative risk of being stunted was 14% lower in children whose mothers used IFA supplements compared to those whose mothers did not use (aRR = 0.86, 95% CI = 0.77–0.97). Additionally, the adjusted relative risk of being stunted was significantly reduced by 23% when antenatal IFA supplementation was started ≤6 months with ≥90 IFA supplements used during pregnancy (aRR = 0.77, 95% CI = 0.64–0.92). Antenatal IFA supplementation significantly reduced the risk of stunting in Nepalese children age <2 years. The greatest impact on the risk reduction of child stunting was when IFA supplements were started ≤6 months with ≥90 supplements were used.

## 1. Introduction

Globally, 45% of under-five deaths can be attributed to child undernutrition [1]. Low birth-weight (birth-weight <2500 grams) and stunting (height-for-age-Z score ≤−2) are major public health problems worldwide [1,2]. Each year an estimated 20 million children are born with low birth-weight, of which 11 million are from South-central Asia (including Afghanistan, Bangladesh, Bhutan, India, Maldives, Nepal, Pakistan and Sri Lanka) [2]. The primary causes of low birth-weight are preterm birth, and/or intrauterine growth restriction [3]. Child stunting reflects linear growth failure due to poor nutrition and/or frequent infections before and after birth [4]. Early childhood stunting leads to poor cognitive, motor and socioeconomic development [4]. In 2011, an estimated 165 million children <5 years of age were affected by stunting, accounting for 17% of total under-five deaths [1]. The largest number of stunted children were in South-central Asia, where 69 million children (36% of under-five population in the region) were stunted [1]. In Nepal, about 12% of newborns have a low birth-weight and 51% of children <5 years of age are stunted [5].

Maternal anaemia in the first or second trimester of pregnancy increases the risk of prematurity and low birth weight [6]. Globally, it is estimated that nearly one-fifth of pregnant women have iron deficiency anaemia at some stage during their pregnancy [1]. In Nepal, nearly a half of pregnant women are anaemic [5]. Research shows that daily use of iron supplements during pregnancy significantly reduces the prevalence of maternal anaemia [6,7,8]. The World Health Organization (WHO) guidelines recommend a daily dose of 30–60 mg iron and 400 µg folic acid supplements throughout pregnancy [9]. In Nepal, iron-folic acid (IFA) supplements are distributed to pregnant women through health facilities and community health workers. During the last decade, Nepal has reported a substantial increase in the coverage of antenatal IFA supplementation, from 23% in 2001 [10] to 80% in 2011 [5].

Studies have documented a significant positive association between the use of IFA supplements during pregnancy and birth-weight [6,7,8]. Two follow-up studies of cluster randomized controlled trials from China [11] and Nepal [12] have evaluated the long term impact of IFA supplements, IFA plus zinc supplements, and multiple micronutrient supplements on the anthropometric status of children. However, trial from China showed no significant differences in the childhood stunting between IFA supplementation and multi-micronutrients during pregnancy [11]. A study from Bangladesh reported a significantly higher prevalence of childhood stunting in children whose mother used multi-micronutrients compared to IFA supplementation during pregnancy [13]. Hence, the effect of antenatal IFA supplementation on child growth is still unclear. In this study, we investigated the impact of antenatal IFA supplementation on childhood stunting in a cohort of Nepalese children <2 years of age.

## 2. Methods

### 2.1. Study Design

The impact of exposure on the outcomes was evaluated using a retrospective cohort study design. Birth history information collected from representative samples of women of reproductive age in Nepal in 2001, 2006 and 2011 allowed us to identify cohorts of women from their most recent live birth within two years prior to interview. The antecedent exposure of the use of antenatal IFA supplements was collected retrospectively by maternal recall. The primary outcomes, low height-for-age in children less than 2 years of age, and maternally perceived small birth size, were measured at the same time the birth history information was recorded. Although the information on exposures and outcomes was collected at the same time, an important element of timing (a retrospective measurement of the use of IFA supplements prior to birth and onset of stunting) allowed causal inference to be assessed. The recall of the exposure was unlikely to be influenced by child’s birth size or stunting status since these data were collected as part of a multi-component survey covering various aspects of women’s reproductive and child health. Furthermore, the outcome status of the child would not be apparent to either the mother or interviewer as it required subsequent data processing.

### 2.2. Data Sources

We used the data on women’s most recent live births within 2 years prior to the date of interview from three Nepal Demographic and Health Surveys (NDHS) 2001 [10], 2006 [14], and 2011 [5]. The de-identified data are available in the public domain from the DHS Measure website (http://dhsprogram.com/). NDHS collects information about socio-demographic, health and nutrition indicators for a nationally representative sample of households through multistage cluster probability sampling [15]. The sampling process has been described in detail elsewhere [5,10,14]. To summarize, enumeration areas, defined as a ward in rural areas and a sub-ward in urban areas, were selected using probability-proportional-to-size sampling. A fixed number of households in each area was randomly selected and all ever-married women of reproductive age (15–49 years) were interviewed in each selected household [5]. All information was self reported. The birth history listed all of the woman’s live-births in chronologic order and contained information about the date of the birth of each child, the singleton or multiple status, sex, and survival status on the day of interview [5,10,14]. For the most recent live-birth during the last 5 years prior to interview, information regarding the use of antenatal care, delivery and postnatal care services was recorded [5,10,14]. Anthropometric data were collected from all children <5 years of age listed in the Household Questionnaire in the NDHS 2001 and the 2006 [10,14]. However, in the NDHS 2011, anthropometric data were collected from children <5 years of age in a subsample of households (*n* = 2582) selected for the men questionnaire [5].

**Figure 1:**
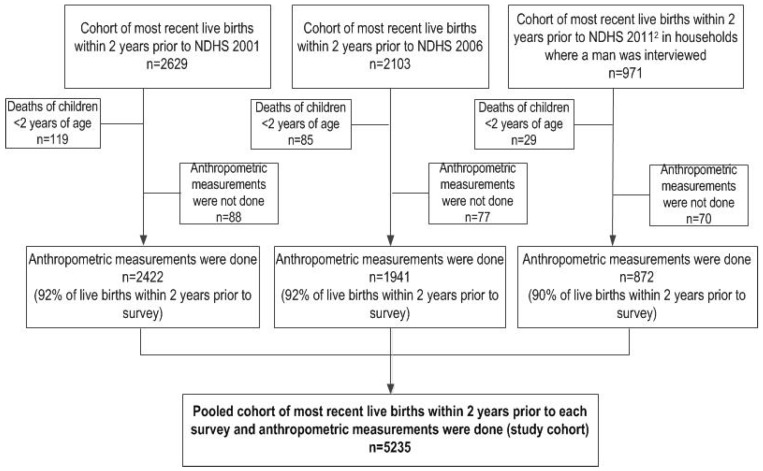
Selection of cohort of the most recent live births within 2 years prior to the interview date from three NDHS^1^ [5,10,14]; ^1^ NDHS: Nepal Demographic and Health Survey; ^2^ In NDHS 2011, the total number of most recent births in the last 2 years prior to the survey was 1980.

For the current study, we examined cohorts of women of reproductive age and the child from their most recent live birth 2 years prior to interview in the 2001 NDHS (*n* = 2629), the 2006 NDHS (*n* = 2103), and the 2011 NDHS (*n* = 971). Anthropometric measurements were not available for children <2 years of age who had died (*n* = 233); these children, and children <2 whose anthropometric measurements were not collected at the time of survey (*n* = 235) were not included in our analysis. Hence, our analytical sample comprises the 5235 most recent live births 2 years prior to interview whose anthropometric measurements were collected (Figure 1). Sample weights were applied to account for the multistage cluster sampling. We selected the most recent live births within the last 2 years to limit recall bias, particularly for maternal use of antenatal care services and maternal perceived birth size.

### 2.3. Ethics

Informed consent was obtained from each respondent in the NDHS and these surveys were approved by the Nepal Health Research Council and ICF Macro Institutional Review Board, Maryland, USA. The current analysis was approved by the Human Research Ethics Committee of the University of Sydney.

### 2.4. Study Outcomes

The primary outcome was low height-for age (stunting) of the child measured at the time of the survey. Height-for-age was the difference between the child’s measured height and the mean height of healthy children in the same age and sex group, expressed as the number of standard deviations or Z-score. A child was classified as being stunted if he/she had a height-for-age Z-score (HAZ) < −2. HAZ (continuous variable), severe stunting (defined as HAZ < −3) and maternal perception of birth size, based on maternal recall, were the secondary outcomes. The question for the maternal perception of birth size in the NDHS was: “When (NAME) was born, was he/she very large, larger than average, average, smaller than average, or very small?” We pooled categories to form a binary variable as “larger than or equal to the average birth size” (including large, larger than average, and average) and “smaller than the average birth size” (including very small and smaller than average). Birth-weight was recorded in only 15% of our sample. We considered maternal perception of birth size a proxy for birth-weight [16].

### 2.5. Exposure Variables and Potential Confounding Factors

The main exposure variable was maternal use of IFA supplements during her last pregnancy within the last 2 years prior to the interview date. In NDHS, the interviewers asked the following questions: “During this pregnancy, were you given or did you buy any iron/folic acid tablets?” and “During the whole pregnancy, for how many days did you take the tablets?” A mother was classified as using antenatal IFA supplements if she reported taking supplements for at least a day during pregnancy. The effect of total number of IFA supplements used during pregnancy on primary outcomes was also assessed. Reports of use of >240 supplements (*n* = 14) were considered as implausible and were excluded from the analysis. In addition, we considered that the maximum number of supplements, that could be used, assuming once daily, were 150, 120, 90, 60 and 30 supplements if a woman started in 5th, 6th, 7th, 8th, and 9th month of pregnancy, respectively. Records with reported number higher than the maximum number that could be consumed when started in 5th to 9th months of pregnancy were also excluded (*n* = 192). To evaluate the impact of the timing of initiation of supplements on the study outcomes, the time of the first antenatal care examination was considered as a surrogate for the start of supplements. As anaemia during first or second pregnancy trimester significantly increases the risk of low birth-weight [6], we categorised the timing of start of supplements as at or before 6 months, and after 6 months during pregnancy. Furthermore, to investigate the combined effect of initiation and total number of supplements used on study outcomes, a variable was constructed using the timing of start of supplements and the total number of supplements used.

We assessed 24 potential confounding factors, classified as community-level and socioeconomic status; maternal and child characteristics; and perinatal health care services (Table 1). To adjust for altitude, a combined variable was used, which reflected median altitude of the ecological regions (Terai, Hill and Mountain) with place of residence (urban and rural areas). Median altitude for the terai urban region was 138 meters; terai rural region was 123 meters; hill urban region was 943 meters; hill rural region was 1252 meters; mountain urban region was 1210 meters; and the mountain rural region was 1563 meters. In addition, we adjusted results for the year of survey (2001, 2006 and 2011), and for the duration of recall *i.e.*, duration between date of birth of a child and date of interview).

**Table 1:** Operational definition and categorization of the potential confounding variables used in the analysis.

Variables	Definition and Categorization
**Community-Level and Socioeconomic Status Factors**	
Ecological region and place of residence	Ecological regions and place of residence of the respondent (1 = Terai region, urban; 2 = Terai region, rural; 3 = Hill region, urban; 4 = Hill region, rural; 5 = Mountain region, urban; and 6 = Mountain, rural).
Maternal marital status	Marital status of the mother (1 = currently married; and 2 = formerly married).
Maternal religion	Religion of the mother (1 = Hindu; 2 = Buddhist and others).
Maternal educational status	Maternal level of attained education (1 = secondary and above; 2 = completed primary; and 3 = no education).
Maternal occupation	Maternal employment status in the past 12 months prior to interview (1 = not working; 2 = working in agriculture; and 3 = working in non-agriculture).
Paternal educational status	Paternal level of attained education (1 = secondary and above; 2 = completed primary; and 3 = no education).
Paternal occupation	Paternal employment status in the past 12 months prior to interview (1 = working in non-agriculture; and 2 = working in agriculture).
Fuel used for cooking	Fuel used for cooking at home (1 = natural gas; and 2 = biomass energy)
Source of drinking water	Source of water used for drinking at home was classified based on WHO/UNICEF guidelines [17] (1 = improved; and 2 = unimproved).
Sanitation facilities	Sanitation refers to toilet facility at home was classified based on WHO/UNICEF guidelines [17] (1 = improved; and 2 = unimproved).
Pooled household wealth index	Composite index of household amenities using pooled NDHS data and a principal component analysis [18] of household assets. The wealth index was used to rank households across the 3 surveys into quintiles.
**Maternal and child characteristics**	
Maternal age at childbirth	Maternal age at childbirth (1 ≤ 20 years; 2 = 20–24 years; and 3 ≥ 25 years).
Maternal desire for pregnancy	Maternal intention to become pregnant (1 = wanted then; 2 = wanted later; and 3 = wanted no more).
Maternal smoking status	Current smoking status of mothers (1 = non-smokers; and 2 = smokers).
Maternal height	Height of mothers at the time of survey (1 = normal height, *i.e.*, ≥145 cm; and 2 = short stature *i.e.*, <145 cm).
Maternal perception of birth size	Subjective assessment of the respondent on the birth size (1 = average; 2 = very small; 3 = smaller than average; 4 = larger than average; and 5 = large).
Birth status	Birth status of the child (1 = singleton; 2 = multiple).
Birth rank and birth interval	Birth rank and birth interval of child (1 = 2nd or 3rd birth rank, birth interval >2 years; 2 = 1st birth rank; 3 = 2nd or 3rd birth rank, birth interval ≤2 years; 4 = ≥4th birth rank, birth interval >2 years; and 5 = ≥4th birth rank, birth interval ≤2 years).
Sex of child	Sex of the child (1 = male; and 2 = female).
Timing of initiation of breastfeeding	Timing of initiation of breastfeeding (1 = <1 h; 2= 1 to 24 h; 3 = >24 h; and 4 =never breastfed).
Duration of breastfeeding	Median duration of breastfeeding in months (as continous variable)
Age of child	Age of children in months (as continuous variable)
Child had diarrhoea during the last 2 weeks prior to interview	Child had diarrhoea within 2 weeks prior to the interview date (1 = no; and 2 = yes).
**Perinatal health services variable**	
Number of antenatal care (ANC) visits	Number of antenatal care visits (1 = no antenatal care visit; 2 = <4 antenatal care visits; and 3 = ≥4 antenatal care visits).

### 2.6. Statistical Analysis

All analyses were undertaken using “svy” commands from Stata 13.1 (Stata-Corp, College Station, TX, USA) to adjust for cluster sampling. Frequencies with weighted percentages were calculated. Poisson regression was used to analyse the study outcomes, except for HAZ, where linear regression was used. Univariate regression models were fitted for each potential factor. We used a multistage approach to determine the final multivariate regression models using a hierarchical approach [19]. At the first stage, all community-level and socioeconomic status variables were included and backward elimination was used to remove non-significant factors. At the second stage, maternal and child characteristics were assessed together with community-level and socioeconomic status variables that were significantly associated with outcomes. At the third stage, number of antenatal care visits was assessed with the significant factors from the previous two steps. At the final stage, exposure variables were included separately with other significant variables mentioned above. A significance level of 0.05 was used, except for duration of recall and year of survey, which were retained in all models. We had a study power of 80% to detect a difference in childhood stunting of 10% between children 2 years of age whose mother did or did not use IFA supplements during pregnancy.

The Population Attributable Risk (PAR) was calculated to assess the risk of child stunting in the population of children 2 years of age that was attributable to women who did not use IFA supplements during pregnancy, and who did not start at or before 6 months and did not use ≥90 supplements during pregnancy. We assumed that the association between IFA supplementation and child’s stunting was causal and that removal of supplementation had no effect on the distribution of other risk factors. The following formula was used to calculate PAR [20,21,22].

PAR = Pe × [(aRR-1)/aRR]

where, Pe was the proportion of children with stunting and aRR was the adjusted relative risk for stunting associated with a mother who did not use IFA supplements, or who did not start at or before 6 months and did not use ≥90 supplements. Based on PAR estimates, the population of children < 2 years in Nepal [23], and the rate of stunting in children < 2 years of age, we estimated the annual number of cases of stunting that could be averted. Nevertheless, the estimates based on PAR calculations depend on the prevalence of the exposure variable, which might vary across populations even within the same country.

## 3. Results

The distribution of community-level, socioeconomic status, maternal and child characteristics, and maternal antenatal care visits is presented in Table 2. About 61% of mothers and 45% of fathers had no education. Nearly three-quarters of mothers reported using improved drinking water and 30% of mothers reported using improved sanitation facilities at home. Nearly one-fifth of mothers were smokers at the time of the survey and 14% were short stature (height < 145 cm). About a quarter of children had a history of diarrhoea during the 2 weeks prior to the interview date.

**Table 2:** Prevalence of community-level, socioeconomic status, maternal and child characteristics and maternal antenatal care visits of the study cohort in Nepal.

	Study Cohort (NDHS 2001–2011)		
Variables	*n* ^1^	*n* ^2^	% ^2^
**Year of Survey**			
2001	2422	2452	47.1
2006	1941	1839	35.3
2011	872	916	17.6
**Community-level and socioeconomic factors**			
**Ecological region and area of residence**			
Terai region, urban	443	231	4.4
Terai region, rural	1973	2461	47.3
Hill region, urban	315	209	4.0
Hill region, rural	1683	1897	36.4
Mountain region, urban	46	5	0.2
Mountain region, rural	775	405	7.7
**Maternal marital status**			
Currently married	5206	5175	99.4
Formerly married	29	32	0.6
**Maternal religion**			
Hindu	4470	4377	84.0
Buddhist and others	765	830	16.0
**Maternal educational status **			
Secondary and above	1175	1159	22.2
Completed primary	891	900	17.3
No education	3169	3148	60.5
**Maternal occupation**			
Not working	1052	1200	23.0
Working in agriculture	3829	3673	70.5
Working in non-agriculture	353	333	6.4
Missing	1	1	0.1
**Paternal educational status**			
Secondary and above	973	950	48.2
Completed primary	1962	1927	37.0
No education	2300	2330	44.8
**Paternal occupation**			
Working in non-agriculture	2814	2820	54.2
Working in agriculture	2266	2229	42.8
Missing	155	158	3.0
**Fuel used for cooking**			
Natural gas	468	465	8.9
Biomass energy	4375	4309	82.8
Missing	392	433	8.3
**Source of drinking water**			
Improved	3777	3847	73.9
Unimproved	1065	926	17.8
Missing	393	434	8.3
**Sanitary facilities**			
Improved	1689	1584	30.4
Unimproved	3154	3190	61.3
Missing	392	433	8.3
**Pooled household wealth index**			
Quintile 1 (Wealthiest)	727	711	13.6
Quintile 2	874	934	17.9
Quintile 3 (Middle)	957	966	18.6
Quintile 4	989	957	18.4
Quintile 5 (Poorest)	1290	1199	23.0
Missing	398	440	8.5
**Maternal and child characteristics**			
**Maternal age at child birth**			
<20 years	3030	3050	58.6
20 to 24 years	1878	1839	35.3
≥25 years	327	318	6.1
**Maternal desire for pregnancy**			
Wanted then	3403	3390	65.1
Wanted later	812	817	15.7
Wanted no more	1020	1000	19.2
**Maternal smoking status**			
Non-smokers	4221	4252	81.7
Smokers	1014	955	18.3
**Maternal height**			
Normal height (≥145 cm)	4523	4477	86.0
Short stature (<145 cm)	712	730	14.0
**Birth status**			
Singleton	5200	5174	99.4
Multiple	35	33	0.6
**Birth rank and birth interval**			
2nd/3rd birth rank, >2 years interval	1650	1623	31.2
1st birth rank	1469	1494	28.7
2nd/3rd birth rank, ≤2 years interval	557	558	10.7
≥4th birth rank, >2 years interval	1196	1179	22.6
≥4th birth rank, ≤2 years interval	363	353	6.8
**Sex of child**			
Male	2595	2583	49.6
Female	2640	2624	50.4
**Timing of initiation of breastfeeding**			
Never breastfed	11	11	0.2
<1 hours	3013	2863	55.0
1 to 24 hours	1169	1098	21.1
>24 hours	1041	1234	23.7
Missing	1	1	0.0
Duration of breastfeeding (months) ^4^	21.0 (12.0, 23.0)		
**Age of children (months) ^4^**	11.6 (±0.1)		
**Child had diarrhoea during the last 2 weeks prior to interview**			
No	3978	3960	76.1
Yes	1257	1247	23.9
**Perinatal health services**			
**Number of antenatal care visits**			
No antenatal care visit	1805	1775	34.1
<4 antenatal care visits	1973	2023	38.8
≥4 antenatal care visits	1457	1410	27.1

^1^ Unweighted; ^2^ Weighting was applied to compensate for the multistage cluster sampling design; ^3^ Median (Interquartile Range); ^4^ Mean (± SD).

Table 3 presents the prevalence of the study exposure and outcome variables. About 51% of mothers did not use IFA supplements during their last pregnancy while 23% of mothers used ≥90 supplements throughout pregnancy. About 44% of mothers started supplements at or before 6 months during their pregnancy. About 23% of mothers started supplements at or before 6 months and took ≥90 supplements during pregnancy. Stunting (HAZ < −2) was present in 31% and severe stunting (HAZ < −3) was present in 10% of children <2 years of age at the time of interview. The rate of stunting and severe stunting in children <2 years of age reduced from 35% and 12% in 2001 to 22% and 6% in 2011, respectively. One fifth of the mothers perceived their child’s birth size as “smaller than the average birth size”. The percentage of children perceived as “smaller than average birth size” declined from 23% in 2001 to 17% in 2011.

Factors associated with stunting in Nepalese children <2 years of age, presented in Table 4, were: living in either hill rural region or mountain rural areas, mothers who had no education, belonging to the poorest pooled household wealth index quintile, mothers who were smokers, maternal short stature, perceived birth size as very small or smaller than average, multiple births, and infants with ≥4th birth rank and birth interval of ≤2 years. Children with large or larger than average birth size, initiated breastfeeding <1 h, Buddhist or other religion, and whose mother had ≥4 antenatal care visits had significantly lower relative risks of stunting.

**Figure 2:**
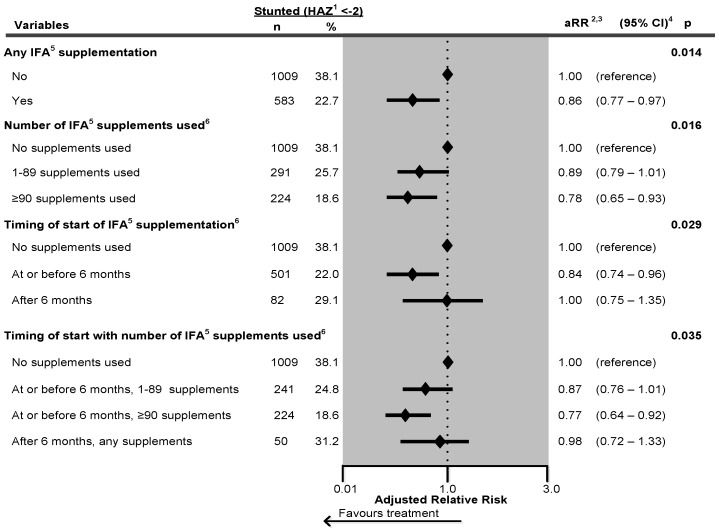
Effect of any iron-folic acid (IFA) supplementation, number of supplements used, timing of start of supplements and a combination of timing of start with number of supplements used on child stunting in Nepal, adjusted Poisson regression. 645 missing values were excluded from the analysis. ^1^ Height-for-Age Z-score; ^2^ Adjusted for ecological region and area of residence, maternal marital status, maternal religion, maternal educational status, maternal occupation, paternal educational status, paternal occupation, fuel used for cooking, source of drinking water, sanitation facilities, pooled household wealth index, maternal age at childbirth, maternal desire for pregnancy, maternal smoking status, maternal height, maternal perception of birth size, birth status, birth rank and birth intervals, sex of baby, timing of initiation of breastfeeding, duration of breastfeeding, age of child, and child had diarrhoea during last 2 weeks prior to interview. In addition, the model was adjusted for year of survey and duration of recall bias. ^3^ aRR: Adjusted relative risk; ^4^ CI: Confidence interval; ^5^ IFA: Iron-folic acid; ^6^ Chi-square test for trend analysis: *p* < 0.0001.

Figure 2 shows a forest plot of the effect of IFA supplementation on child stunting in Nepal. The adjusted relative risk of being stunted was 14% lower among children whose mother used any IFA supplements during pregnancy compared to those whose mothers used no supplementation (aRR = 0.86, 95% CI = 0.77–0.97). Children whose mothers used ≥90 supplements during pregnancy had a 22% significantly lower adjusted relative risk of stunting by (aRR = 0.78, 95% CI = 0.65–0.93) compared to those whose mother did not use supplements. Children whose mother started the supplements at or before 6 months during pregnancy had a 16% significantly lower adjusted relative risk of stunting (aRR = 0.84, 95% CI = 0.74–0.96) compared to those whose mother did not use supplements. When the supplements started at or before 6 months with ≥90 supplements were consumed during pregnancy, the adjusted relative risk of being stunted was significantly lower by 23% (aRR = 0.77, 95% CI = 0.64–0.92) compared to those whose mother did not use supplements.

**Table 3:** Prevalence of study exposure and outcome variables of the study cohort and by year of survey in Nepal.

	Study Cohort			2001	2006	2011
Variables	*n* ^1^	*n* ^2^	% ^2^	% ^2^	% ^2^	% ^2^
**Study exposure variables**						
**Iron-folic acid (IFA) supplementation**						
No	2675	2645	50.8	75.8	34.5	16.5
Yes	2560	2562	49.2	24.2	65.5	83.5
**Number of iron-folic acid (IFA) supplements used**						
No IFA supplementation	2675	2645	50.8	75.8	34.5	16.5
1–89 supplements used	1142	1133	21.8	16.3	30.3	19.3
≥90 supplements used	1212	1206	23.1	5.6	30.0	56.4
Missing	206	223	4.3	2.3	5.2	7.7
**Timing of start of iron-folic acid (IFA) supplements during pregnancy**						
No IFA supplementation	2675	2645	50.8	75.8	34.5	16.5
At or before 6 months	2289	2280	43.8	19.9	58.9	77.4
After 6 months	271	282	5.4	4.3	6.6	6.1
**Timing of start with number of iron-folic acid (IFA) supplements used during pregnancy**						
No IFA supplementation	2675	2645	50.8	75.8	34.5	16.5
At or before 6 months, 1–89 supplements used	980	971	18.6	13.8	25.8	17.4
At or before 6 months, ≥90 supplements used	1212	1206	23.2	5.6	30.0	56.5
After 6 months, any IFA supplements used	162	162	3.1	2.5	4.4	1.9
Missing	206	223	4.3	2.3	5.2	7.7
**Study outcome variables**						
**Stunting status of child**						
Not stunted	3603	3616	69.4	64.7	71.6	77.8
Stunted (HAZ ^3^ < −2)	1632	1591	30.6	35.3	28.4	22.2
**Stunting status of child**						
Not severely stunted	4706	4691	90.1	87.6	91.6	93.5
Severely stunted (HAZ ^3^ <-3)	529	516	9.9	12.4	8.4	6.5
**Maternal perception of birth size**						
Average or larger than average	4080	4157	79.8	77.3	81.7	82.7
Smaller than average	1154	1047	20.1	22.7	18.3	17.0
Missing	1	3	0.1	0.0	0.0	0.3

^1^ Unweighted; ^2^ Weighting was applied to compensate for the multistage cluster sampling design; ^3^ HAZ: Height-for-Age Z-score.

**Table 4:** Factors associated with stunting in Nepal, unadjusted and adjusted Poisson regression.

	Stunted (HAZ ^1^ < −2)		Unadjusted			Adjusted ^2^		
Variables	*n*	%	RR ^3^	95% CI ^4^	*p*	RR ^3^	95% CI ^4^	*p*
**Community-level and socioeconomic factors**								
**Ecological region and place of residence**					**<0.0001**			**0.001**
Terai region, urban	43	18.7	1.00	(reference)		1.00	(reference)	
Terai region, rural	681	27.7	1.48	(1.18–1.85)		1.22	(0.96–1.54)	
Hill region, urban	30	14.3	0.76	(0.53–1.09)		0.91	(0.66–1.26)	
Hill region, rural	661	34.8	1.86	(1.49–2.32)		1.34	(1.05–1.70)	
Mountain region, urban	1	4.6	0.24	(0.04–1.52)		0.32	(0.06–1.77)	
Mountain region, rural	176	43.5	2.32	(1.82–2.96)		1.58	(1.22–2.03)	
**Maternal religion**					**0.251**			**0.011**
Hindu	1359	30.1	1.00	(reference)		1.00	(reference)	
Buddhist and others	232	27.9	0.90	(0.75–1.08)		0.83	(0.72–0.96)	
**Maternal educational status**					**<0.0001**			**0.001**
Secondary and above	183	15.8	1.00	(reference)		1.00	(reference)	
Completed primary	230	25.6	1.62	(1.30–2.00)		1.26	(0.99–1.61)	
No education	1177	37.4	2.36	(1.98–2.81)		1.47	(1.18–1.83)	
**Pooled household wealth index**					**<0.0001**			**0.015**
Quintile 1 (Wealthiest)	98	13.8	1.00	(reference)		1.00	(reference)	
Quintile 2	219	23.4	1.70	(1.31–2.21)		1.22	(0.92–1.62)	
Quintile 3 (Middle)	302	31.3	2.27	(1.76–2.92)		1.44	(1.08–1.93)	
Quintile 4	358	37.4	2.72	(2.15–3.43)		1.52	(1.14–2.05)	
Quintile 5 (Poorest)	521	43.5	3.15	(2.50–3.98)		1.60	(1.18–2.17)	
**Maternal and child characteristics**								
**Maternal smoking status**					**<0.0001**			**0.006**
Non-smokers	1163	27.4	1.00	(reference)		1.00	(reference)	
Smokers	428	44.8	1.64	(1.49–1.80)		1.14	(1.04–1.25)	
**Maternal height**					**<0.0001**			**<0.0001**
Normal stature (≥145 cm)	1274	28.5	1.00	(reference)		1.00	(reference)	
Short stature (<145 cm)	317	43.5	1.52	(1.37–1.70)		1.37	(1.23–1.52)	
**Maternal perception of birth size**					**<0.0001**			**<0.0001**
Average	892	28.9	1.00	(reference)		1.00	(reference)	
Very small	123	44.0	1.52	(1.30–1.78)		1.29	(1.10–1.52)	
Smaller than average	328	42.8	1.48	(1.31–1.67)		1.22	(1.09–1.37)	
Larger than average	208	23.5	0.81	(0.69–0.96)		0.80	(0.70–0.92)	
Large	39	20.8	0.72	(0.52–0.99)		0.61	(0.43–0.85)	
**Birth status**					**<0.0001**			**0.038**
Singleton	1570	30.3	1.00	(reference)		1.00	(reference)	
Multiple	21	63.6	2.09	(1.61–2.72)		1.49	(1.02–2.18)	
**Birth rank and birth interval**					**<0.0001**			**0.005**
2nd/3rd birth rank, >2 years interval	446	27.5	1.00	(reference)		1.00	(reference)	
1st birth rank	377	25.2	0.92	(0.80–1.06)		1.05	(0.91–1.22)	
2nd/3rd birth rank, ≤2 years interval	165	29.5	1.07	(0.91–1.27)		1.07	(0.90–1.26)	
≥4th birth rank, >2 years interval	421	35.7	1.30	(1.14–1.48)		1.06	(0.95–1.20)	
≥4th birth rank, ≤2 years interval	183	51.9	1.89	(1.65–2.17)		1.30	(1.13–1.49)	
**Timing of initiation of breastfeeding**					**0.200**			**0.016**
Never breastfed	6	50.3	1.00	(reference)		1.00	(reference)	
<1 hours	845	29.5	0.59	(0.31–1.11)		0.42	(0.22–0.80)	
1 to 24 hours	343	31.3	0.62	(0.33–1.17)		0.44	(0.23–0.84)	
>24 hours	397	32.2	0.64	(0.34–1.21)		0.49	(0.25–0.93)	
**Perinatal health services**								
**Number of antenatal care visits**					**<0.0001**			**0.024**
No antenatal care visit	699	39.4	1.00	(reference)		1.00	(reference)	
<4 antenatal care visits	631	31.2	0.79	(0.71–0.88)		1.01	(0.92–1.12)	
≥4 antenatal care visits	260	18.5	0.47	(0.40–0.55)		0.81	(0.68–0.97)	

645 missing values were excluded from the analysis. ^1^ HAZ: Height-for-Age Z-score; ^2^ Adjusted for ecological region and place of residence, maternal marital status, maternal religion, maternal educational status, maternal occupation, paternal educational status, paternal occupation, fuel used for cooking, source of drinking water, sanitation facilities, pooled household wealth index, maternal age at childbirth, maternal desire for pregnancy, maternal smoking status, maternal height, maternal perception of birth size, birth status, birth rank and birth intervals, sex of baby, timing of initiation of breastfeeding, duration of breastfeeding, age of child, child had diarrhoea during 2 weeks prior to survey, and number of antenatal care visits. In addition, the model was adjusted for year of survey and duration of recall bias. ^3^ RR: Relative risk; ^4^ CI: Confidence interval.

As the use of antenatal care services and IFA supplementation variables were correlated in our sample, therefore, we constructed a combined variable of use of other antenatal care services and IFA supplementation. Children whose mother used IFA supplements with or without use of other antenatal care services had lower adjusted relative risk of being stunted by 12% (aRR = 0.88, 95% CI = 0.76–0.99) compared to children whose mother did not use IFA or any other antenatal care services.

The adjusted risk of being severely stunted in Nepalese children <2 years was significantly reduced by 37% (aRR = 0.63, 95% CI = 0.49–0.82) in children whose mother used IFA supplements compared to those whose mother did not use IFA supplements during pregnancy.

Our estimates showed that 3.8% (95% CI = 0.26%–9.25%) of stunting could be attributed to non-use of antenatal IFA supplements in Nepalese children <2 years of age. With universal coverage of IFA supplements, stunting could be averted in 7500 Nepalese children <2 years of age annually. Similarly, we found that 8.4% (95% CI = 0.2%–18.4%) of stunting was attributed to not starting supplements at or before 6 months and not taking ≥90 supplements during pregnancy. If all Nepalese women started supplements at or before 6 months and consumed ≥90 supplements during pregnancy, stunting could be averted in 16800 Nepalese children <2 years of age per annum.

The factors associated with the smaller than average birth size in Nepal, presented in Table S1 (Supplementary data), were: mothers had no education, belonging to the poorest pooled household wealth index quintile, biomass energy used for cooking at home, mothers who were smokers, multiple births and female babies. Figure S1 (Supplementary data) showed that with maternal antenatal IFA supplementation, the adjusted risk of smaller than average birth size significantly reduced by 19% (aRR = 0.81, 95% CI = 0.70–0.95) compared to no maternal antenatal IFA supplementation. The adjusted risk of smaller than average birth size significantly reduced by 21% (aRR = 0.79, 95% CI = 0.64–0.97) in children whose mother used ≥90 IFA during pregnancy compared to those whose mother did not use supplements. The adjusted risk of smaller than average birth size significantly reduced by 22% (aRR = 0.78, 95% CI = 0.63–0.96) in children whose mother started the IFA at or before 6 months and took ≥90 supplements during pregnancy compared to those whose mother did not use supplements.

The findings of adjusted linear regression analysis for HAZ are presented in Table S2 (Supplementary data). The adjusted mean ±SD HAZ was significantly higher (−1.14 ± 0.47) in children whose mother used any IFA supplements compared to children whose mother did not use IFA supplements (−1.70 ± 0.43). When started supplements ≤6 months and took ≥90 supplements during pregnancy, the adjusted mean (±SD) HAZ was significantly higher, (−0.97 (± 0.45)) compared to children whose mother did not use IFA supplements (−1.70 (± 0.43)).

## 4. Discussion

### 4.1. Main Findings and Their Significance

We found that maternal use of IFA supplements during pregnancy significantly reduced the adjusted risk of stunting by 14% compared to mothers who never used supplements in Nepalese children <2 years of age. Further, the adjusted risk of being severely stunted was significantly reduced by 37% with use of antenatal IFA supplements in children <2 years of age. For children whose mother used ≥90 supplements the adjusted risk of being stunted was reduced by 22%. Children whose mother initiated IFA supplements at or before 6 months during pregnancy the adjusted risk of being stunted was reduced by 16%. With initiation of supplements at or before 6 months and use of ≥90 supplements during pregnancy, the adjusted risk of being stunted was reduced by 23% and these children had a higher adjusted mean HAZ. With universal coverage to start IFA supplements at or before 6 months and use of ≥90 IFA supplements during pregnancy, stunting could be averted in 16800 Nepalese children <2 years of age each year.

To our knowledge, the current study is the first adequately powered study to report a protective effect of antenatal IFA supplementation on child stunting in Nepal at national level. Our findings provide important evidence for policy-makers and program managers in developing countries dealing with maternal, newborn and child health, nutrition and supplementation programs. In Nepal, like many other developing countries, IFA supplements are given to pregnant women at or after 4 months of gestation. We found that a greater preventive effect of IFA supplementation on stunting was seen when supplements started at or before 6 months with use of ≥90 supplements during pregnancy. Similarly starting supplementation earlier in pregnancy and sustaining it throughout pregnancy has the greatest effect on reducing early neonatal mortality in Nepal and Pakistan [24]. However, there is a need to conduct prospective trials with long term follow-up time periods to confirm the effect of maternal use of IFA supplements on survival and growth of infants in developing countries.

### 4.2. Strengths and Limitations

An important strength of the current study was the appropriate timing progression in the data used for the analyses, in which the main study exposure, the use of IFA supplements during pregnancy was measured by maternal recall and was antecedent to the study outcomes. This timing sequence allows causal inference although the retrospective design is not as strong as a prospective cohort design. To minimise recall bias [25,26,27], we selected live births 2 years prior to the interview date and also adjusted our models for duration of recall. Use of supplements is likely to be recalled adequately, but recall of number of supplements and starting data may have been less accurate as seen with heaping of number of supplements used around intervals of 30. As suggested by others [16], we used maternal perception of birth size as a proxy for birth-weight. To increase validity, we performed multivariate models adjusted for several potential confounding factors at community, household and individual-levels, including perceived birth size for stunting models. Moreover, the study had >80% power to show clinical important differences in the risk of childhood stunting between children whose mothers used IFA compared to those who did not use. As childhood stunting is associated with high altitude [28], we adjusted our analyses for ecological regions and found that higher altitude areas were associated with childhood stunting compared to low altitude areas.

One limitation was that use of IFA during pregnancy was based on maternal recall and biochemical indicators of iron status could not be assessed. Maternal recall of number of IFA might not be accurate as reflected in the heaping of data for the number of supplements in intervals of 30 and this may have led to misclassification. Although analyses were adjusted for a number of potential confounding factors, mothers who took IFA were not randomly assigned and there is a possibility of remaining residual confounding. Similarly, blood samples were not taken in the 2001 NDHS and we could not include the child’s anaemia status. In addition, there is only information about consumption of fortified foods in the 24 h preceding the interview. Recent use of fortified foods is not an indicator of how long they have been used. Longer term use would be needed to expect an impact on growth. Hence, we did not adjust the regression models for this indicator. Some variables like maternal and paternal occupation within the last 12 months and maternal smoking status at the time of interview were not infant specific because these only presented the most recent conditions. These limitations may have led to an underestimate/overestimate of the effects of IFA. Our findings should therefore be interpreted with these caveats in mind, although the overall validity of the findings remains unchallenged.

### 4.3. Comparison with Other Studies

Our findings of the effect of IFA supplementation on birth size are consistent with meta-analyses which report 19%–20% reduction in low birth-weight with maternal use of IFA supplements during pregnancy [6,7,8]. A Cochrane review found a significant reduction in the risk of low birth-weight-by 11% and small for gestational age infants by 13% with multiple micronutrients compared to the IFA. However, more evidence is required before changing universal policy for antenatal IFA supplementation with multiple micronutrient supplements [29]. The higher reduction in the risk of low birth-weight by multiple micronutrients compared to IFA could derive from enhanced absorption of iron due to the presence of other micronutrients, such as ascorbic acid in the multiple micronutrients [30].

In contrast to the preventive effect of IFA on childhood stunting in the current study, findings from two follow-up studies of the cluster randomised controlled trials from China [11] and Nepal [12] have shown no significant reduction in the risk of childhood stunting with maternal use of IFA compared to multi-micronutrients and other supplementations. In China the lack of measured effect of IFA on the risk of childhood stunting could have been due to small sample size and inadequate statistical power, the low prevalence of low birth weight and the low rate of stunting [11]. Similarly, in Nepal the small sample size and assessment of older children (6–8 years old) could have influenced the findings [12]. The impact on birth size in our study of early initiation and greater numbers of supplements consumed are consistent with three randomised controlled trials which reported an effect of iron supplementation on birth-weight. Two US trials enrolled women early in their pregnancy (10 and 12 weeks gestation) and reported a significant increase in mean birth weight in infants whose mother took iron supplements compared to controls [31,32]. In contrast, a randomised trial from Australia, where pregnant women were enrolled from 20 weeks of gestation and received either low dose iron (20 mg daily) or placebo reported no difference in birth-weight between the two study groups [33]. The findings from the US [31,32] and evidence from the current study suggest that the effect of IFA on birth-weight could be due to early initiation and appropriate dosage of supplements during pregnancy. Research shows that maternal perception of small birth size [34] and low birth-weight [35] increase the risk of stunting in children. A recent meta-analysis of 19 cohorts (*n* = 44374) from low- and middle-income countries has found that low birth-weight (<2.5 kg within 72 h of birth), small for gestational age (<10th percentile) and prematurity (gestational age <37 weeks) were associated with an increased odds of 2.92 (95% CI = 2.56–3.33), 2.32 (95% CI = 2.12–2.54), and 1.69 (95% CI = 1.48–1.93) for stunting in children 12–60 months of age, respectively. Further, small for gestational age and term infants with low birth-weight had higher odds of childhood stunting (OR = 3.00, 95% CI = 2.36–3.81) compared to small for gestational age and term infants without low birth-weight (OR = 1.92, 95% CI = 1.75–2.11) in low- and middle-income countries [35]. Nutritional interventions, especially IFA supplementation in regions with high maternal anaemia, are important during pregnancy to improve birth size/weight and subsequently reduce stunting.

### 4.4. Impact on Reduction in Stunting

Our PAR analysis showed that 8.5% of stunting was attributed to not starting supplements at or before 6 months and not taking ≥90 supplements during pregnancy. If all pregnant women were to start supplements at or before 6 months and take ≥90 supplements during pregnancy, stunting could be averted in 16800 Nepalese children <2 years of age each year. However, it is important to highlight that PAR estimates depends on the prevalence of exposure which might vary across populations. Nepal in the recent past has improved its IFA supplementation program by introducing a district-level intervention package [36]. However, the variation in coverage of IFA and total number of supplements used across regions and socioeconomic status groups needs to be improved. Recent studies have identified factors associated with non-use of IFA supplements and barriers to IFA supplementation during pregnancy [37,38]. Hence, there is a need to strengthen and modify the current distribution strategies and women should be encouraged to start supplements earlier in pregnancy.

To conclude, we found that use of antenatal IFA supplements significantly reduced the risk of being stunted and severely stunted in Nepalese children <2 years of age. The greatest effect on risk reduction was found with early initiation (at or before 6 months) with use of ≥90 IFA supplements during pregnancy, suggesting a timing- and dose-dependent response. This current evidence from Nepal supports the need to carry out prospective trials in low- and middle-income countries to examine the effect of IFA supplements during pregnancy on childhood stunting.

## Supplementary Materials

Table S1. Factors associated with smaller than average birth size in Nepal, unadjusted and adjusted Poisson regression; Figure S1. Effect of any iron-folic acid (IFA) supplementation, number of supplements used, timing of start of supplements and a combination of timing of start with number of supplements used on smaller than average birth size in Nepal, results of adjusted Poisson regression; and Table S2. Effect of any iron-folic acid (IFA) supplementation, number of supplements used, timing of start of supplements and a combination of timing of start with number of supplements used on height-for-age Z-score in Nepal, results of adjusted linear regression.
