# Supplementary material for: Iron-Folic Acid Supplementation During Pregnancy Reduces the Risk of Stunting in Children Less Than 2 Years of Age: A Retrospective Cohort Study from Nepal
Source: Nutrients. 2016 Jan 27;8(2):67. doi: 10.3390/nu8020067 (PMC4772031; doi:10.3390/nu8020067)
Supplement: Supplementary file 1 [file nutrients-08-00067-s001.doc]

Supplementary Materials: Iron-Folic Acid Supplementation During Pregnancy Reduces the Risk of Stunting in Children Less Than 2 Years of Age: A Retrospective Cohort Study from Nepal

Yasir Bin Nisar, Michael J Dibley and Victor M Aguayo

**Table S1.** Factors associated with smaller than average birth size in Nepal, unadjusted and adjusted Poisson regression.

|  | **Smaller Than Average Birth Size** | | **Unadjusted** | | | **Adjusted ^1^** | | |
| --- | --- | --- | --- | --- | --- | --- | --- | --- |
| **Variables** | **n** | **%** | **RR ^2^** | **95% CI ^3^** | **p** | **RR ^2^** | **95% CI ^3^** | **p** |
| **Community-level and socioeconomic factors** |  |  |  |  |  |  |  |  |
| **Ecological region and area of residence** |  |  |  |  | **<0.0001** |  |  | **0.001** |
| Terai region, urban | 38 | 16.3 | 1.00 | (reference) |  | 1.00 | (reference) |  |
| Terai region, rural | 435 | 17.7 | 1.08 | (0.83–1.42) |  | 0.74 | (0.56–0.98) |  |
| Hill region, urban | 38 | 18.0 | 1.10 | (0.76–1.60) |  | 1.39 | (0.93–2.07) |  |
| Hill region, rural | 418 | 22.0 | 1.35 | (1.03–1.77) |  | 0.87 | (0.65–1.18) |  |
| Mountain region, urban | 1 | 10.4 | 0.64 | (0.35–1.16) |  | 0.62 | (0.32–1.18) |  |
| Mountain region, rural | 118 | 29.2 | 1.78 | (1.31–2.43) |  | 1.07 | (0.77–1.47) |  |
| **Maternal educational status** |  |  |  |  | **<0.0001** |  |  | **0.045** |
| Secondary and above | 156 | 13.5 | 1.00 | (reference) |  | 1.00 | (reference) |  |
| Completed primary | 158 | 17.6 | 1.31 | (1.05–1.63) |  | 1.15 | (0.90–1.46) |  |
| No education | 733 | 23.3 | 1.73 | (1.45–2.06) |  | 1.31 | (1.05–1.64) |  |
| **Fuel used for cooking** |  |  |  |  | **<0.0001** |  |  | **0.023** |
| Natural gas | 59 | 12.7 | 1.00 | (reference) |  | 1.00 | (reference) |  |
| Biomass energy | 923 | 21.4 | 1.69 | (1.27–2.25) |  | 1.48 | (1.06–2.08) |  |
| **Pooled household wealth index** |  |  |  |  | **<0.0001** |  |  | **0.040** |
| Quintile 1 (Wealthiest) | 94 | 13.2 | 1.00 | (reference) |  | 1.00 | (reference) |  |
| Quintile 2 | 148 | 15.8 | 1.19 | (0.90–1.58) |  | 1.03 | (0.74–1.45) |  |
| Quintile 3(Middle) | 188 | 19.5 | 1.47 | (1.12–1.93) |  | 1.20 | (0.83–1.74) |  |
| Quintile 4 | 210 | 21.9 | 1.66 | (1.29–2.14) |  | 1.20 | (0.85–1.72) |  |
| Quintile 5 (Poorest) | 338 | 28.2 | 2.13 | (1.67–2.71) |  | 1.40 | (1.00–1.99) |  |
| **Maternal and child characteristics** |  |  |  |  |  |  |  |  |
| **Maternal smoking status** |  |  |  |  | **<0.0001** |  |  | **0.011** |
| Non-smokers | 772 | 18.2 | 1.00 | (reference) |  | 1.00 | (reference) |  |
| Smokers | 275 | 28.8 | 1.59 | (1.38–1.83) |  | 1.22 | (1.05–1.42) |  |
| **Birth status** |  |  |  |  | **<0.0001** |  |  | **<0.0001** |
| Singleton | 1033 | 20.0 | 1.00 | (reference) |  | 1.00 | (reference) |  |
| Multiple | 15 | 44.7 | 2.24 | (1.44–3.47) |  | 2.56 | (1.68–3.90) |  |
| **Sex of child** |  |  |  |  | **<0.0001** |  |  | **<0.0001** |
| Male | 438 | 17.0 | 1.00 | (reference) |  | 1.00 | (reference) |  |
| Female | 609 | 23.2 | 1.37 | (1.20–1.55) |  | 1.36 | (1.19–1.56) |  |

638 missing values were excluded from the analysis. ^1^ Adjusted for ecological region and area of residence, maternal marital status, maternal religion, maternal educational status, maternal occupation, paternal educational status, paternal occupation, fuel used for cooking, source of drinking water, sanitation facilities, pooled household wealth index, maternal age at childbirth, maternal desire for pregnancy, maternal smoking status, maternal height, birth status, birth rank and birth intervals, and number of antenatal care visits. In addition, the model was adjusted for year of survey and duration of recall bias; ^2^ RR: Relative risk; ^3^ CI: Confidence interval.


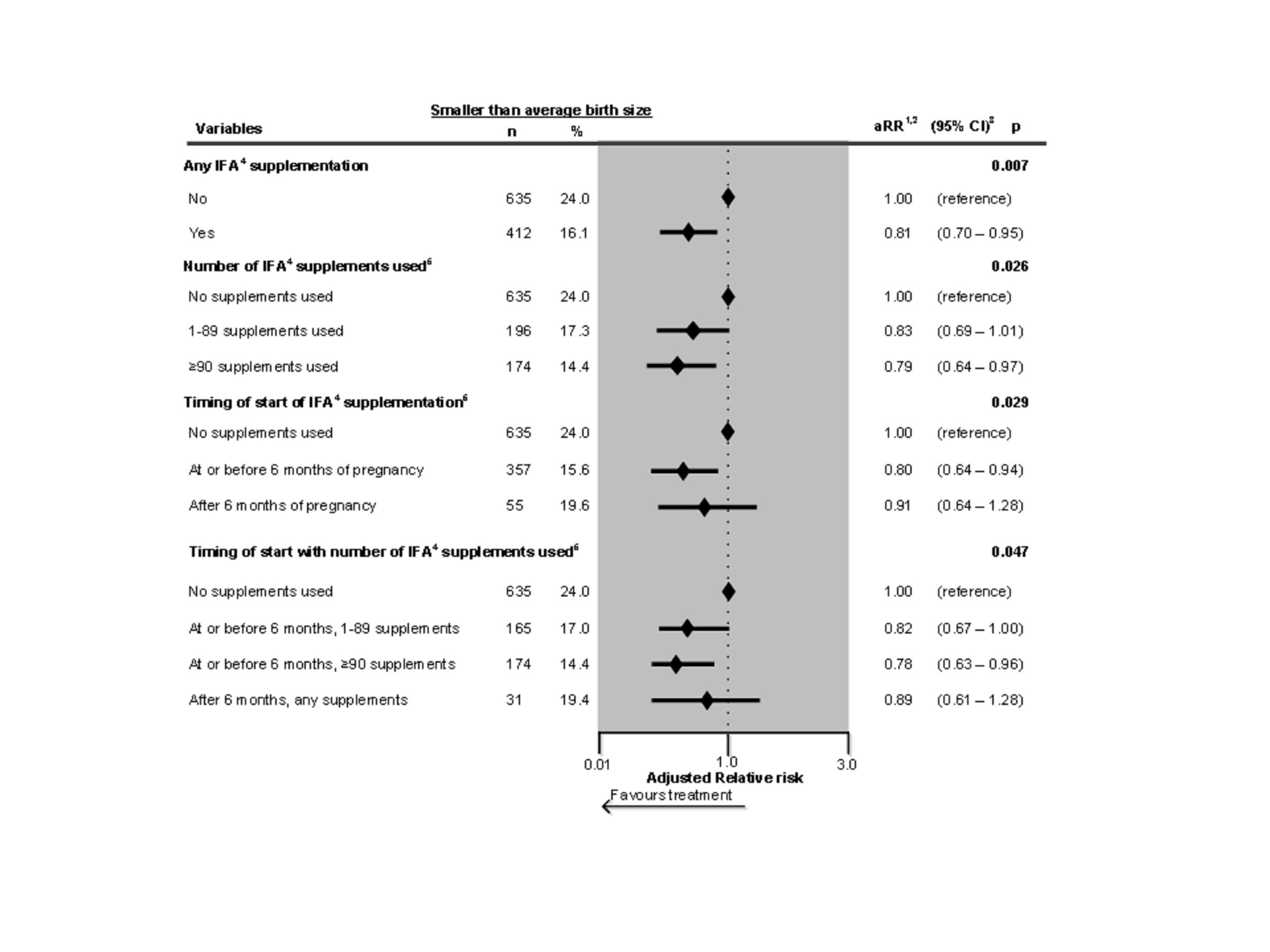


# Figure S1. Effect of any iron-folic acid (IFA) supplementation, number of supplements used, timing of start of supplements and a combination of timing of start with number of supplements used on smaller than average birth size in Nepal, results of adjusted Poisson regression. 638 missing values were excluded from the analysis; ^1^ Adjusted for ecological region and area of residence, maternal marital status, maternal religion, maternal educational status, maternal occupation, paternal educational status, paternal occupation, fuel used for cooking, source of drinking water, sanitation facilities, pooled household wealth index, maternal age at childbirth, maternal desire for pregnancy, maternal smoking status, maternal height, birth status, birth rank and birth intervals, and number of antenatal care visits. In addition, the model was adjusted for year of survey and duration of recall bias; ^2^ aRR: Adjusted Relative risk; ^3^ CI: Confidence interval; ^4^ IFA: Iron-folic acid; ^5^ Chi-square test for trend analysis: *p* < 0.0001.

# Table S2. Effect of any iron-folic acid (IFA) supplementation, number of supplements used, timing of start of supplements and a combination of timing of start with number of supplements used on height-for-age Z-score in Nepal, results of adjusted linear regression

|  | **Adjusted ^1^** | | | |
| --- | --- | --- | --- | --- |
| **Variables** | **Mean (±SD) HAZ ^2^** | **Coefficient** | **95% CI ^3^** | **p** |
| **Any IFA supplementation** |  |  |  | **0.002** |
| No | −1.70 (±0.43) | 1.00 | (reference) |  |
| Yes | −1.14 (±0.47) | 0.13 | (0.05–0.22) |  |
| **Number of IFA supplements used** |  |  |  | **0.002** |
| No supplements used | −1.70 (±0.43) | 1.00 | (reference) |  |
| 1-89 supplements | −1.28 (±0.45) | 0.10 | (0.01–0.20) |  |
| ≥90 supplements | −0.97 (±0.45) | 0.20 | (0.08–0.31) |  |
| **Timing of start of IFA supplementation** |  |  |  | **0.008** |
| No supplements used | −1.70 (±0.43) | 1.00 | (reference) |  |
| At or before 6 months | −1.11 (±0.46) | 0.14 | (0.05–0.22) |  |
| After 6 months | −1.44 (±0.45) | 0.08 | (−0.15–0.30) |  |
| **Timing of start and number  of IFA supplements used** |  |  |  | **0.006** |
| No supplements used | −1.70 (±0.43) | 1.00 | (reference) |  |
| At or before 6 months, 1–89 supplements | −1.26 (±0.44) | 0.10 | (0.01–0.20) |  |
| At or before 6 months, ≥90 supplements | −0.97 (±0.45) | 0.20 | (0.08–0.31) |  |
| After 6 months and any supplements used | −1.40 (±0.47) | 0.10 | (−0.13–0.34) |  |

645 missing values were excluded from the analysis; ^1^ Adjusted for ecological region and area of residence, maternal marital status, maternal religion, maternal educational status, maternal occupation, paternal educational status, paternal occupation, fuel used for cooking, source of drinking water, sanitation facilities, pooled household wealth index, maternal age at childbirth, maternal desire for pregnancy, maternal smoking status, maternal height, maternal perception of birth size, birth status, birth rank and birth intervals, sex of baby, timing of initiation of breastfeeding, duration of breastfeeding, age of child, and child had diarrhoea during last 2 weeks prior to survey. In addition, the model was adjusted for year of survey and duration of recall bias; ^2^ HAZ: Height-for-age Z-score; ^3^ CI: Confidence interval.
